# Antioxidant Capacity Alterations in Bangladeshi Underutilized Fruits: InsightsFrom In Vitro Gastrointestinal Digestion Studies

**DOI:** 10.1155/ijfo/5762710

**Published:** 2026-06-20

**Authors:** Majbaul Alam, Shreef Mahmood, Nusrat Jahan, Towkir Ahmed Ove, Noor Hassan Mohammad Rubel Mozumder, Habiba Khatun

**Affiliations:** ^1^ Department of Food Science and Nutrition, Hajee Mohammad Danesh Science and Technology University, Dinajpur, Bangladesh, hstu.ac.bd; ^2^ Departmentof Horticulture, Hajee Mohammad Danesh Science and Technology University, Dinajpur, Bangladesh, hstu.ac.bd

**Keywords:** antioxidant capacity, bioactive compounds, in vitro digestion, underutilized fruits

## Abstract

The current research investigated effect of in vitro digestion on bioactive compounds and the antioxidant capacity of five underutilized fruits (Amla, Olive, Hog plum, Elephant Apple, and Bilimbi) grown in Bangladesh. In case of fresh extracts, maximum (523.31 mg/100 g fruit weight [FW]) and minimum (45.62 mg/100 g FW) ascorbic acid content was found in amla and olive, respectively. Moreover, the highest total phenol content was found in elephant apple (379.36 mg of GAE/100 FW), whereas the lowest total phenol was found in olive (346.28 mg of GAE/100 g FW). Regarding total flavonoid content (TFC) five fruits differed significantly for both fresh and digested extract. Amla exhibited the highest flavonoid content among the five fruits in both fresh (51 mg QE/100 g) and digested extracts (53 mg QE/100 g). Although TFC increased after in vitro digestion, a decreasing trend was observed in total flavanol content after digestion for all five fruits. The results also showed that DPPH radical scavenging activity significantly reduced after in vitro digestion. The highest DPPH activity was observed in Elephant apple before (92%) and after (46.67%) digestion. Although the ABTS assay (% inhibition) of all fruit extracts decreased, Elephant Apple still showed 89.75% inhibition. However, gastrointestinal in vitro digestions improved FRAP which was highest for Amla 14.68 mg BHT/mL. Therefore, the outcomes of the current study suggest that among the five fruits, Amla, and Elephant apple, which have high ascorbic acid, total flavonoid, and antioxidant capacity, could be considered for nutraceuticals and value‐added products.

## 1. Introduction

Noncommunicable diseases killed almost 43 million people in 2021 of which 77% are from lower or lower middle‐income country [1]. Bangladesh also faces a high prevalence of noncommunicable diseases. Previous research pointed out that free radicals formed in our body due to oxidative stress are responsible for many chronic diseases. The degenerative effect of these free radicals may be easily quenched by the antioxidants, especially polyphenols present in fruits [2]. Moreover, fruits are one of most important groups of our healthy diet due to their various health‐promoting compounds of which vitamins, minerals and antioxidants are of prime importance [3, 4]. Consumption of fruits is associated with lower incidence of overall mortality, cancer, heart disease, hypertension, stroke, eye disease, metabolic disorders like diabetes [5]. Daily consumption of 400 g or five servings of fruits and vegetables could possibly contribute to combat this problem as suggested by WHO/FAO. A small percentage of the population follows this recommendation [6].

Bangladesh has abundant supply of typical fruits in around the year with fertile land and excellent ecosystem for fruits cultivation. These fruits possess functional characteristics such as antioxidant content, anticytotoxicity compound content, antitumor content etc. [7, 8]. Along with the popular seasonal fruits there are many underutilized fruits in Bangladesh such as amla (*Phyllanthus emblica*), elephant apple (*Dillenia indica*) hog plum (*Spondias pinnata*), olive (*Elaeocarpus floribundus*), and bilimbi (*Averrhoa bilimbi*). Most of the underutilized fruits are rich in potent functional components and require minimum management for cultivation. For instance, amla has been reported to contain polyphenols, flavonoids, tannins, and phenolic acids with strong antioxidant potential [9]. Elephant apple (*D. indica*) has been found to have multiple bioactive compounds whereas hog plum (*S. pinnata*), olive (*E. floribundus*), and bilimbi (*A. bilimbi*) were also reportedas potential dietary source of health boosting nutrients like tocopherols, polyphenols, squalene, phytosterols, spondiol, glycospondin, and ascorbic acid [10–13]. Nevertheless, these fruits have received relatively little attention as potential antioxidant sources. They have been neglected due to their low popularity, limited scientific data about their nutritional and physical characteristics, and a lack of promotional activities to promote their nutritional value [14–16].

The functionality of antioxidants depends on their bioavailability after digestion, absorption, and metabolism as the stability of the polyphenols could be affected by the varying pH level and enzymes secreted in the gastrointestinal tract. Moreover, release of carbohydrates, amino acids, acids and other molecules associated with polyphenols during digestion may also alter the antioxidant activity [17–19]. Therefore, the stability of these important antioxidants must be analyzed after digestion (either in vitro or in vivo) to assess their bioavailability.

Most of the research on these fruits primarily concentrated on the proximate composition, biochemical properties and few bioactive compounds while studies on the in‐vitro effect on their antioxidant capacity are limited. For example, methanolic extract of amla has been widely studied and reported for its antioxidant, anticancer, and antidiabetic activity [20]. Additionally, only biochemical properties of several Bangladeshi underutilized fruits including velvet apple (*Diospyros discolor*), river ebony (*Diospyros peregrina*), monkey jack (*Artocarpus lakoocha*), cowa (*Garcinia cowa*), etc have been investigated by Hossain et al. [21]. Thus, the stability and bioavailability of antioxidants or beneficial bioactive compounds of these fruits after gastrointestinal in vitro digestion have not been substantially explored. It is essential to address these gaps through a systematic and digestion‐based framework to establish their true nutritional value and unlock their potential for functional food development and nutrition security. Therefore, in the present study, five underutilized fruits (hog plum, bilimbi, amla, olive, and elephant apple) grown in Bangladesh were selected to explore the stability of bioactive compounds and antioxidant activity of these fruits after in vitro gastrointestinal digestion.

## 2. Materials and Methods

### 2.1. Sample collection

Five fresh mature fruits, that is, amla, elephant apple, hog plum, olive, and bilimbi (Table 1) were collected in mature stage from the research fruits orchard of Hajee Mohammad Danesh Science and Technology University (HSTU) especially from the Department of Horticulture. The maturation was confirmed by trained horticulturist′s visual inspection. After properly washing, peels and seeds of the fruits were discarded. The resulting pulp was used for the preparation of fruit extracts which were used for all the analysis.

**Table 1:** Selected underutilized fruits in Bangladesh.

**Raw fruit image**	**English name**	**Local name (Bengali)**	**Scientific name**	**Family**	**Period of harvesting**
	Amla	Amloki	*Phyllanthus emblica* (syn. Emblica officinalis)	Phyllanthaceae	January 2025
	Elephant apple	>Chalta	*Dillenia indica*	Dilleniaceae	October 2024
	Hog plum	Amra	*Spondias pinnata*	Anacardiaceae	October 2024
	Olive	Jolpai	*Elaeocarpus floribundus*	Elaeocarpaceae	October 2024
	Bilimbi	Bilimbi	*Averrhoa bilimbi*	Oxalidaceae	September 2024

### 2.2. Chemicals and Standards

Sodium hydroxide (NaOH), phenolphthalein, methanol, sodium‐potassium tartarate, 3,5‐dinitro salicylic acid (DNS), Na_2_SO_3_, phenol, glucose, Folin–Ciocalteu reagent (FCR), gallic acid, quercetin, metaphosphoric acid, EDTA, sulfuric acid (H_2_SO_4_), ammonium molybdate, HCl, aluminim chloride (AlCl_3_), sodium chloride (NaCl), DPPH (2,2‐diphenyl‐1‐picrylhydrazyl), sodium acetate, trolox, trichloroacetic acid, ferric chloride (FeC1_3_), potassium ferricyanide (K_3_Fe (CN)_6_),green vitriol (FeSO_4_.7H_2_O), PBS (phosphate buffer saline), ABTS (2,2‐azinobis 3‐ethyl benzothiazoline‐6‐sulfonic acid), potassium chloride (KCl), BHT (butylated hydroxytoluene), pepsin preparation, pancreatin preparation, bile preparation, sodium bicarbonate (NaHCO_3_), and so on. All of these reagents and chemicals were of analytical grade and were procured from renowned manufacturers like Merck, Germany; Sigma–Aldrich, Steinheim, Germany.

### 2.3. Biochemical Analysis of Selected Fruits

Titrable acidity and pH of the selected five fruits were evaluated by following the standard methods suggested by Tyl et al. [22]. Titrable acidity was measured by using NaOH in the presence of phenolphthalein (2–3 drops) indicator until pink color was developed. pH value of the selected sample was measured by using a digital pH meter (HANNA, pH 211 Microprocessor pH meter, Romania) by performing two‐point (buffer 4.0 and 7.0) calibrations.

### 2.4. Determination of Bioactive Compound Content of Selected Fruits

#### 2.4.1. Preparation of Fruit Extracts for Flavonol, Flavonoid, ABTS, DPPH, and Ferric‐Reducing Antioxidant Power (FRAP) Assay

Five fruits extract preparation was accomplished as per the method proposed by Kumar et al. [23] protocol with little modifications. These extracts were used for evaluating flavonol, flavonoid content, and antioxidant capacity assessment. At first, 25 ml of methanol was added with 3 g of fresh fruit tissue and homogenized using homogenizer (Model‐ VCR 3, VELP Scientifica, Italy). Then, the homogenates were kept at 4°C for 12 h followed by a centrifugation at a rate of 4000 rpm for 30 minutes. Finally, the supernatants were collected and stored at −80°C until the further analysis.

#### 2.4.2. Determination of Total Ascorbic Acid Content

Total ascorbic acid content of the selected five fruit samples was determined using the spectrophotometric procedure suggested by Hossain et al. [24] with few modifications. Ten grams of fruit was homogenized with 100 mL oxalic acid‐EDTA solution in a homogenizer (VELP Scientifica, Italy) and subsequently centrifuged for 10 minutes at 3000 rpm and filtered. A 5 mL aliquot from the supernatant was then taken in a 25 mL volumetric flask in which 1 mL 5% H_2_SO_4_ solution, 0.5 ml metaphosphoric acid‐acetic acid solution and 2 ml of 5% ammonium molybdate reagent were added. Afterward, the mixture was made to a volume of 25 mL with addition of distilled water and incubated for 15 min and finally the absorbance was measured at 760 nm with a UV‐VIS spectrophotometer.

#### 2.4.3. Estimation of Total Polyphenol Content

Total phenol compound in the fruit samples was quantified using Folin–Ciocalteu (FC) reagent and the spectrophotometric method recommended by Ove et al. [25] and Podloucká et al. [26] with minor alterations. Firstly, 1 g fruit flesh mixed with 4 mL methanol (80% aqueous) containing 2.7% HCl subjected to homogenization and centrifugation. Afterward, 300 *μ*L of the sample extract was added with 2.25 mL Folin–Ciocalteu reagent which further followed by addition of 2.25 mL of sodium carbonate solution (60 g/L). After that, the mixture was vortexed and incubated at room temperature for 90 min. Finally, the absorbance was taken at 765 nm via using a UV‐VIS spectrophotometer (PG Instrument Ltd. Model T60, United Kingdom). The phenol content was then estimated using the standard curve generated by utilizing gallic acid and the results were presented as the milligrams of gallic acid equivalents (GAE)/100 g FW.

#### 2.4.4. Estimation of Total Flavonol Content

Total flavonol content of the selected fresh fruits as well as in the digested solution was determined according to the procedure described by Omoruyi et al. [27] with slight modifications. At first, 2 mL of the sample extract was mixed with 2 mL of AlCl_3_ prepared in 2% ethanol. Afterward, 3 mL of sodium acetate solution (50 g/L) was added to the mixture and incubated at 20°C for 2.5 hours. Finally, the absorbance was measured at 440 nm in a UV‐VIS spectrophotometer (PG instruments T60, United Kingdom). Quercetin was used as a standard for the total flavonol content calculation and results were expressed as microgram of quercetin equivalent (QE)/100g FW.

#### 2.4.5. Determination of Total Flavonoid Content (TFC)

The TFC of selected fruits was quantified by a method suggested by Toh et al. [28]. An aliquot of 2 mL extract was mixed with 0.2 mL of 0.5% sodium nitrate solution and incubated for 5 minutes. After the incubation 0.2 mL of 10% aluminum chloride was added to the mixture and allowed to stand for 6 minutes. After that, 2 mL of 1 M NaOH was added with the mixture solution. Then, the final volume of the mixture was made up to 5 mL with 80% methanol and mixed thoroughly. Lastly, using a UV‐VIS spectrophotometer, absorbance of the mixture was recorded at 510 nm and results were expressed as milligram QE/100 g FW. Standard curve was developed using Quercetin (0–0.2 mg/mL concentration) solutions.

### 2.5. Determination of Antioxidant Capacity

#### 2.5.1. ABTS Assay

ABTS assay of the selected samples was performed by following method suggested by Wang et al. [29] with minor modifications. ABTS cation radical stock solution was prepared by mixing ABTS solution with a potassium persulfate solution. The mixture was then maintained in dark room at room temperature for 16 hours before use. The fresh ABTS working solution was prepared by diluting the stock solution with phosphate buffer solution (PBS) at pH 7.4 to achieve an absorbance of 0.40 at 734 nm. An aliquot of 100 *μ*L of diluted sample extract was mixed with 1900 *μ*L of ABTS working solution. Then, absorbance was measured using spectrophotometer (Model T60, PG Instrument Ltd., United Kingdom) at 734 nm. Standard curve was developed by using 100 *μ*L of trolox solution (1.5 mM) prepared by dilution with PBS. The final result was expressed in % inhibition unit.

#### 2.5.2. FRAP Assay

The Ferric reducing power of the selected fruits extract was estimated by following the method of Ainunnisa et al. [30] with slight adjustments. Firstly, 1 mL fruit extract was added to the solution containing phosphate buffer and (K_3_Fe (CN)_6_) (1% w/v). Then, the mixture was incubated at 50°C for 30 minutes. After incubation, it was mixed with 2.5 mL trichloroacetic acid solution (10% w/v) and centrifuged (10 minutes at 3000 rpm). An aliquot of 2.5 mL from the supernatant solution was then taken and mixed with 0.5 mL of FeCl_3_ (0.1%, w/v) and 2.5 mL distilled water. Finally, the absorbance was recorded at 700 nm with a UV‐VIS spectrophotometer against blank sample. BHT was utilized to construct a standard curve and values were expressed as mg BHT/mL of sample.

#### 2.5.3. DPPH Radical Scavenging Activity

DPPH free radical scavenging activity of the selected fruits extracts was analyzed following the method suggested by Bhardwaj et al. [31]. An aliquot of the fruit extract (0.1 mL) was added to 2.9 mL of DPPH solution (containing 0.025 g/L in methanol) and incubated in dark for 30 minutes. The absorbance of the resultant solution was measured at 515 nm. The standard curve for this assay was also constructed using trolox.

### 2.6. Gastrointestinal in Vitro Digestion

Crude extracts of five selected fruits were subjected gastrointestinal in vitro digestion according to the methodology suggested by Pavan et al. [32] with little alterations. Two ml of crude fruit extract was mixed in a saline solution (prepared by mixing 5 mM KCl, 150 mM BHT, and 140 mM NaCl) at a ratio of 1:4 v/v (sample/saline solution). Afterward, the saline‐sample mixture was agitated for 10 minutes at 21°C in a shaking incubator (Vision Scientific, VS‐8480SN, South Korea). Then, thorough addition of 0.1 M/1 M HCl, the mixture was acidified to pH 2.0. When the pH 2.0 was achieved, 0.25 mL pepsin solution (containing 0.2 g pepsin/5 mL 0.1 M HCl) was added to it and incubated for 1 hr at 37°C in a shaking incubator. After the completion of gastric digestion, the intestinal digestion was started by the addition of 1 M NaHCO_3_/0.1 M NaHCO_3_ until the target intestinal pH 6.9 was achieved. Then, 1.25 mL of pancreatic and bile mixed solution (containing 0.225 g bile extract and 0.0375 g pancreatin in a volume of 18.75 mL 0.1 M NaHCO_3_) was added followed by the incubation with shaking at 37^o^C for 2 hr. The ultimate volume of the digested sample by addition of brine was made up to 14 mL. This digested sample was subjected to flavonoids, flavanol, DPPH, ABTS, and FRAP assay.

### 2.7. Statistical Analysis

For each experiment (both before and after in vitro digestion), single factor analysis of variance (ANOVA) was performed for all variables using the statistical software SPSS (Version 22). All analyses were conducted in triplicates. The results were presented as mean ± SE (standard error). One way ANOVA test was conducted to compare the bioactive compounds, total flavonoid content, flavanol content, and antioxidant capacity among the five fruit species. Paired comparison *t-*test was accomplished to check the change between fresh/undigested and digested extract for the same fruit. All analyses were considered as significant at *p* ≤ 0.05.

## 3. Results and Discussion

### 3.1. Biochemical Properties

Results of biochemical properties of the five fruits presented in Table 2 showed that acidity and pH varied largely among five fruits extract. pH value of the pulp of selected fruits showed a significant difference (*p* < 0.05) ranging from 2.79 ± 0.006 for amla (lowest) to 4.96 ± 0.006 for olive (highest), (Table 2). The present result is accordance with the findings of Parveen et al. [33] who found pH value 2.82 and 2.84 in “Kanchan” and “Banarasi” amla variety, respectively.

**Table 2:** Biochemical properties of selected five fruits.

**Sample**	**pH**	**Titrable acidity(%)**
Amla	2.79 ± 0.006^e^	1.81 ± 0.023^a^
Elephant apple	3.20 ± 0.012^c^	0.36 ± 0.020^d^
Hog plum	2.85 ± 0.012^d^	0.49 ± 0.020^c^
Olive	4.96 ± 0.006^a^	0.75 ± 0.023^b^
Bilimbi	3.88 ± 0.006^b^	0.34 ± 0.020^d^

*Note:* Results are expressed as mean ± SE. ^a–e^ indicate significant difference among the fruit samples (*p* < 0.05).

Moreover, Table 2 shows that the titrable acidity of the selected fruits varied from 0.34 ± 0.020 to 1.81 ± 0.023*%*. Highest value of titrable acidity 1.81% was found from amla followed by olive (0.75%) and hog plum (0.49%), and lowest acidity was found in elephant apple and bilimbi (Table 2). The titratable acidity of amla (*P. emblica*) in the present study showed close agreement, with minor variations, compared to the values reported by Golap et al. [24] for Kanchan (1.90%), NA‐7 (1.64%), and Indian desi (2.08%) varieties. [34]. Lowest value found in bilimbi (0.434%) coincided with little variation with the result reported by Soumya and Bindu [35].

### 3.2. Bioactive Compound Content

#### 3.2.1. Total Phenol Content (TPC) (milligrams of GAE/100 g FW)

The TPC was significantly (*p* < 0.05) diverse among the fruit samples (Table 3). The phenolic content of the selected fruits ranged from 346.28 to 379.36 mg of GAE/100 g FW. The results presented in Table 3, indicated that elephant apple (379.36 mg of GAE/100 g FW) contained highest TPC among the sample and olive scored the lowest (346.28 ± 0.54). Current research findings were almost similar with the TPC of Indian amla (339.0 ± 6.0 mg of GAE/100 g) and olives (299.35 ± 48.65 mg of GAE/100 g) found by other researchers [36, 37], except for hog plum which showed different values. The variation in hog plum may have originated from different maturation levels, growing conditions. Based on prior investigation, in the case of olive, different cultivars contain high polyphenol content and several bioactive compounds like oleuropein, hydroxytyrosol, and tyrosol in early stage of maturation but these levels decrease during fruit development and maturation [38]. Furthermore, presence of some nonphenolic compounds such as vitamins and minerals in the extracts that can react with the Folin–Ciocalteu reagent may lead to overestimation [39].

**Table 3:** Expression of total phenol content (TPC) and ascorbic acid content of selected five fruits.

**Sample**	**TPC (mg of GAE/100 g FW)**	**Ascorbic acid (mg/100 g FW)**
Amla	351.57 ± 0.11^cd^	523.31 ± 0.81^a^
Elephant apple	379.36 ± 0.61^a^	106.38 ± 0.19^b^
Hog plum	370.79 ± 0.17^ab^	60.72 ± 0.97^c^
Olive	346.28 ± 0.54^d^	45.62 ± 0.65^e^
Bilimbi	363.70 ± 0.30^bc^	51.59 ± 1.78^d^

*Note:* Results are represented mean ± SE. ^a–e^ indicate significant difference among the fruit samples (*p* < 0.05).

Abbreviation: FW, fruit weight.

#### 3.2.2. Total Ascorbic Acid (mg/100 g FW)

Ascorbic acid or vitamin C is a powerful water‐soluble antioxidant. The vitamin C content among the five fruits was differed significantly (p < 0.05) and ranged from 45.62 to 523.31 mg per 100 g FW. The maximum amount of vitamin C was found in amla (523.31 mg/100 g FW) and it was followed by elephant apple (106.38 mg/100 g FW), hog plum (60.72 mg/100 FW), and bilimbi (51.59 mg/100 g FW) whereas lowest vitamin C content was found in olive (45.62 mg/100 g FW) (Table 3). The ascorbic acid content reported for the local (desi) amla variety—ranging from 486 to 480.20 mg per 100 g fresh weight—by Singh et al. [40] and Bakshi et al. [34] was slightly lower than the values observed in our present study. Variation in ascorbic acid content may originate from physicochemical changes associated with the degree of maturation. Research on *Spondias mangifera* (Indian hog plum) demonstrated that ascorbic acid accumulates during fruit development, which is an important indicator of fruit maturation [41]. During these changes, degradation of some organic acid may also lower the acidity through increasing pH. This happens may be due to conversion of starch into simple sugar during maturation that will finally increase total soluble solid content [42].

### 3.3. Effect of In Vitro Digestion on TFC and Flavonol Content

**Figure 1:**
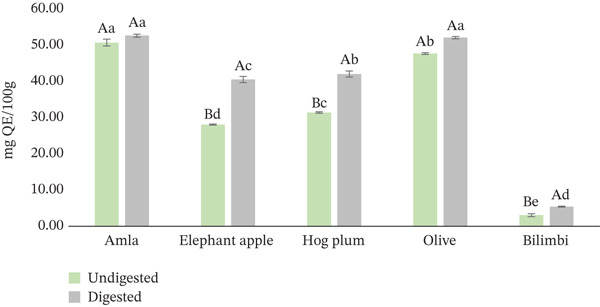
Effect of in vitro digestion on total flavonoid content of fresh and digested fruit extracts. *Note:* Values are expressed as mean ± SE. a–e (lowercase), means followed by different alphabets are significantly different among samples (*p* < 0.05). A and B (uppercase), means followed by different alphabets are significantly different among the undigested and digested extract of the same fruit (*p* < 0.05). Abbreviation: QE, quercetin equivalent.

Flavonoids possess strong antioxidant activities and are widely present in fruits and vegetables and exhibit multiple positive biological effects such as free radical‐scavenging activity. It might be through direct ROS (reactive oxygen species) scavenging or hindering ROS generation through the inhibition of enzymes or chelation of trace elements involved with free radical generation [43]. A significant (*p* < 0.05) difference among the samples was observed in both undigested and digested stages as shown in Figure 1. Amla differed from the others by presenting the highest flavonoid content (51 ± 0.007 and 53 ± 0.003 mg QE/100 g) in both undigested or fresh and digested condition, respectively. All fruit samples showed a significant (*p* < 0.05) increase in the TFC after gastrointestinal in vitro digestion (Figure 1). Similar increasing trends was also observed by in papaya and Brazilian uvaia (*Eugenia pyriformis* Cambess) fruits as well as in amla and tomato pomace extracts after in vitro digestion [33, 44, 45]. The increment of flavonoid content might be occurred due to the release of complex or bound flavonoids from the fruit matrix due to the chemical changes in flavonoids during digestion process such as enzymatic and acidic activity, action of bile salts, and change in redox potential during digestion [44, 46, 47].

**Figure 2:**
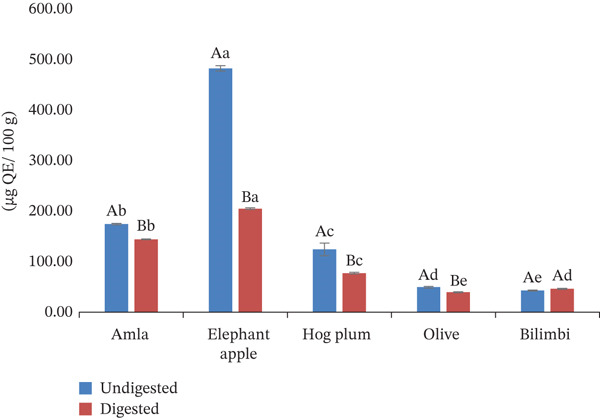
Effect of in vitro digestion on flavonol content of fresh and digested fruit extracts. *Note:* Values are expressed as mean ± SE. a–e (lowercase), means followed by different alphabets are significantly different among samples (*p* < 0.05). A and B (uppercase), means followed by different alphabets are significantly different among the undigested and digested extract of the same fruit (*p* < 0.05). Abbreviation: QE, quercetin equivalent.

According to the results presented in Figure 2, mean values of flavonol content ranged from 42.99 ± 1.06 to 482.34 ± 6.81 *μ*g QE/100 g for undigested samples and from 39.61 ± 1.07 to 204.84.±2.15 *μ*g QE/100 g for digested extracts. This indicates that the flavonol content is significantly reduced after digestion. Further, the data shows that the elephant apple had highest flavanol whereas bilimbi had lowest flavanol for both undigested and digested extracts. The results were in accordance with Sultana and Anwar [48] who stated similar result (359.4 mg QE/100 g) for mulberry fresh extract.

This study has demonstrated that flavonol content was significantly (*p* < 0.05) decreased during a simulated digestion except for bilimbi. This decrease in flavonol content might be explained by the high reactivity of antioxidants at the gastric phase with very high acidic condition [49–51]. Thus, when antioxidants are exposed to such conditions, they can alter into different compounds showing dissimilar structural forms as well as different chemical properties. The methods used for antioxidants analysis are highly sensitive to structural forms. Therefore, there might be some antioxidants which are not detected due to their structural deformities, but they still have the ability to donate hydrogen and scavenge free radicals [52].

### 3.4. Effect of In Vitro Digestion on Antioxidant Capacity of Fresh and Digested Fruits Extracts

#### 3.4.1. DPPH (% Inhibition) Assay

**Figure 3:**
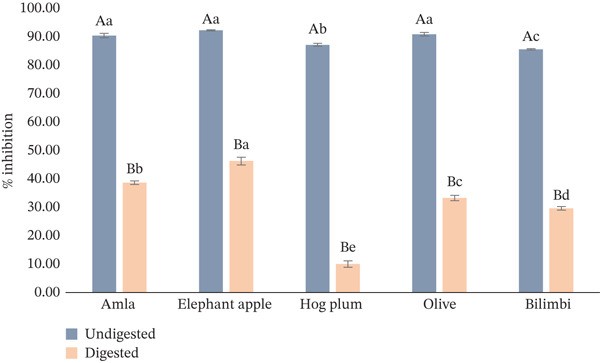
Effect of in vitro digestion on DPPH assay (% inhibition) of fresh and digested fruit extracts. *Note:* Values are expressed as mean ± SE. a–e (lowercase), means followed by different alphabets are significantly different among samples (*p* < 0.05). A and B (uppercase), means followed by different alphabets are significantly different among the undigested and digested extract of the same fruit (*p* < 0.05).

In the DPPH assay, antioxidants react with DPPH^•^ radical by donating hydrogen and reduced it from violet colored DPPH^•^ to the yellow‐colored DPPH. The degree of the discoloration exhibits the free radical quenching potential of the sample [53]. The results showed that DPPH % inhibition value of the fruits ranged from 85.58 ± 0.24 to 92.25 ± 0.20*%* and 10.0 ± 1.14 to 46.25 ± 1.34*%* in undigested and digested fruit extracts, respectively (Figure 3). Furthermore, it has also been revealed that among the undigested fruit extracts, amla, elephant apple, and olive did not differ significantly. However, all these three extracts had significant (*p* < 0.05) difference when compared to bilimbi and hog plum regarding DPPH % inhibition. Similar DPPH inhibition value (30.5%–84.8%) was also observed in pomegranate juice [52]. Regarding the digested extract, a significant (*p* < 0.05) difference was also revealed among the fruit samples. Decreasing trend in DPPH^•^ scavenging activity was observed in digested extracts (Figure 3). Similar results of decreasing DPPH values after intestinal digestion for olive pomace and jucara fruits were also reported by Alhuthayli et al. [54] and Schulz et al. [55]. This could be due to the structural degradation or oxidation of the polyphenols in alkaline pH in the intestine. Moreover, the phenolic compounds can be polymerized and form complex compounds in the intestinal environment which eventually have less access to DPPH reagents [54–56].

#### 3.4.2. ABTS^•+^ Radical Scavenging Activity

**Figure 4:**
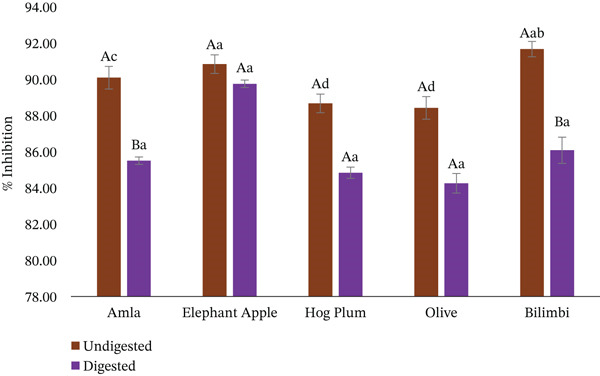
Effect of in vitro digestion on ABTS assay (% inhibition) of fresh and digested fruit extracts. *Note:* Values are expressed as mean ± SE. a–d (lowercase), means followed by different alphabets are significantly different among samples (*p* < 0.05). A and B (uppercase), means followed by different alphabets are significantly different among the undigested and digested extract of the same fruit (*p* < 0.05).

This assay works on the principle of scavenging of the relatively stable blue colored ABTS^**•+**^ cation radical solution converting it into a colorless stable ABTS product solution. The degree of decolorization represents the amount of ABTS^**•+**^ that has been scavenged. In the current study, percentage inhibition of the fruit extract against the ABTS^•+^ radical cation was quantified. Results showed that the overall % inhibition of ABTS radical ranged from 88.42 ± 0.62 to 91.67 ± 0.42*%* in undigested extracts (Figure 4). Bilimbi displayed the highest values (91.67 ± 0.42*%* inhibition) followed by elephant apple (90.83 ± 0.51*%* inhibition), amla (90.08 ± 0.62*%* inhibition), hog plum (88.67 ± 0.51*%* inhibition), and olive (88.42 ± 0.62*%* inhibition) in undigested or fresh extracts.

After in vitro digestion, the inhibition values of amla and bilimbi (*p* < 0.05) decreased significantly. The % inhibition of ABTS radical in digested extracts ranged from 84.25 ± 0.54 to 89.75 ± 0.20*%*. However, bilimbi showed the highest value among digested samples. The reduction in percent inhibition may occur due to the degradation or transformation of the polyphenols in digestion medium [53] as mentioned earlier. Our findings are in line with the results reported by Li et al. [57] and Li et al. [58] who reported that ABTS scavenging activity drastically decreased after simulated gastrointestinal digestion of red‐fleshed apples and fermented kiwifruit extracts, respectively. However, scavenging activity of fermented dragon fruit–kiwi beverages and date fruit extracts increased during or after digestion, possibly due to the release of bound phenolics or transformation into more active forms [59, 60].

#### 3.4.3. FRAP Assay

**Figure 5:**
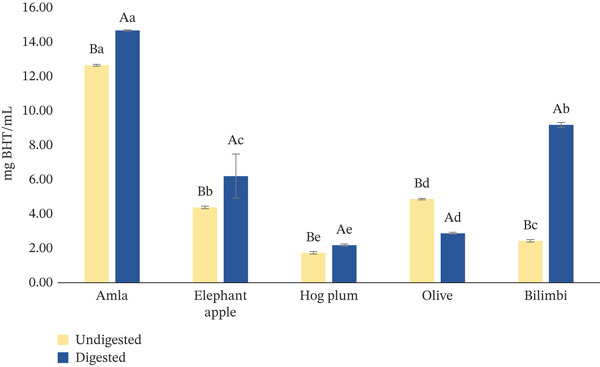
Effect of in vitro digestion on ferric reducing power of fresh and digested fruit extracts. *Note:* Values are expressed as mean ± SE. a–e (lowercase), means followed by different alphabets are significantly different among samples (*p* < 0.05). A and B (uppercase), means followed by different alphabets are significantly different among the undigested and digested extract of the same fruit (*p* < 0.05).

The FRAP values of the undigested fruit extracts varied significantly (*p* < 0.05) among the fruits that ranged from 1.74 ± 0.07 to 12.66 ± 0.06 mg BHT/mL (Figure 5). Among the samples, amla exhibited the highest FRAP value (12.66 ± 0.06 mg BHT/mL), followed by elephant apple (4.39 ± 0.07 mg BHT/mL), bilimbi (2.24 ± 0.07 mg BHT/mL), olive (2.88 ± 0.06 mg BHT/mL), and hog plum (1.74 ± 0.07 mg BHT/mL).

After in vitro digestion, FRAP values for all fruit samples significantly (*p* < 0.05) increased (Figure 5). After digestion, the maximum value (14.68 ± 0.04 mg BHT/mL) was also exerted by amla whereas hog plum showed the minimum (2.19 ± 0.07 mg BHT/mL) value. Similar increasing trend of FRA*P* value after intestinal digestion was found in pineapple and pomegranate juice [61]. Increment in FRAP value after digestion might be due to breaking down of fruit matrix and easy release of phenolic acids and flavonoids, breakdown of more complex polyphenols like proanthocyanidins or tannins into simpler phenolic acids and flavonoids, degradation of nonantioxidant compounds like lipid or carbohydrates that have the ability to interact with antioxidants and thus enhance the FRAP value, different enzyme assisted hydrolysis, and isomerization of antioxidants during digestion [44, 62, 63].

## 4. Conclusions

Although substantial research has been conducted on the common fruits in Bangladesh, several underutilized fruits are yet to be explored in terms of their bioactive compounds and antioxidant capacity. The present study examined the effect of in vitro gastrointestinal digestion on the bioactive compounds and antioxidant capacity of five underutilized fruits grown in Bangladesh. The findings of the current study show that, the aforementioned fruits have different biochemical and bioactive compounds. From the biochemical parameter viewpoint, amla showed to have maximum value of titrable acidity and vitamin C compared to the other four fruits. Among the five fruits, amla and elephant apple were found to be superior to hog plum, bilimbi and olive in terms of the bioactive compound content and antioxidant capacity for both undigested and digested extract. Furthermore, in vitro digestion showed significant (*p* < 0.05) difference in the flavonol and TFC as well as on antioxidant capacity. Total flavonoids content and FRAP value increased after in‐vitro digestion whereas ABTS and DPPH value either remained similar or decreased for all the fruits samples. Among the five fruits, amla and elephant apple may be considered as the potential sources of antioxidant capacity and bioactive contents. However, this research could act as a preliminary screening for the investigated fruit. Future research could aim for human or other trial animal system (in vivo) along with toxicity analysis to explore the exact scenario within the living organism.

## Author Contributions

Majbaul Alam: conceptualization, methodology, investigation, data curation, writing original draft, reviewing and editing. Shreef Mahmood: supervision, methodology, formal analysis, writing—reviewing and editing. Nusrat Jahan: methodology, investigation, data curation. Towkir Ahmed Ove: writing—reviewing and editing. Noor Hassan Mohammad Rubel Mozumder: writing—reviewing and editing. Habiba Khatun: conceptualization, methodology, supervision, writing—reviewing and editing.

## Funding

No funding was received for this manuscript.

## Ethics Statement

This study does not involve any human or animal study.

## Conflicts of Interest

The authors declare no conflicts of interest.

## Data Availability

Data will be provided by the corresponding author upon request.
